# HOMEFREE: Scaling‐Up Evidence‐Based Guidelines for IV‐to‐Oral Antibiotic Switch in Hospitalised Children, an Implementation Study Protocol

**DOI:** 10.1111/jpc.70528

**Published:** 2026-08-05

**Authors:** Jessica Suna, Daniel Tanti, Adam Bartlett, Christopher Blyth, Karin Thursky, Evette Buono, Lolita Java, Gabrielle Haeusler, Penelope Bryant, Lisa Hall, Gregory Merlo, Brendan McMullan

**Affiliations:** ^1^ University of Queensland Brisbane Australia; ^2^ Research Division CQUniversity Rockhampton Australia; ^3^ University of New South Wales Sydney Australia; ^4^ Sydney Children's Hospital, Randwick Sydney Australia; ^5^ Wesfarmers Centre of Vaccines & Infectious Diseases, The Kids Research Institute Australia and School of Medicine University of Western Australia Nedlands Australia; ^6^ Department of Infectious Diseases Perth Children's Hospital Nedlands Australia; ^7^ Department of Microbiology, PathWest Laboratory Medicine QEII Medical Centre Nedlands Australia; ^8^ University of Melbourne Parkville Australia; ^9^ National Centre for Antimicrobial Stewardship Melbourne Australia; ^10^ Peter MacCallum Cancer Centre Melbourne Australia; ^11^ Clinical Excellence Commission St Leonards Australia; ^12^ Murdoch Children's Research Institute Parkville Australia; ^13^ Royal Children's Hospital Parkville Australia; ^14^ Clinical Excellence Queensland Herston Australia

## Abstract

**Background:**

Around half of children admitted to hospital receive intravenous (IV) antibiotics, contributing to higher costs, vascular access complications and longer hospital stays. A single‐site pilot in a tertiary Australian paediatric hospital implemented nationally endorsed Australasian Stewardship of Antimicrobials in Paediatrics (ANZPID‐ASAP) IV‐to‐oral antibiotic switch guidelines, improving timely switch rates from 64% to 82%. Following this success, a broader study is planned to assess the feasibility of scaling this approach across hospitals of varying types and sizes within NSW, Australia.

**Aims:**

The HOMEFREE study hypothesises that implementation of an IV‐to‐oral antibiotic switch programme based on endorsed guidelines and supported by audit and feedback will increase the proportion of hospitalised children transitioned to oral therapy or antibiotic cessation within 24 h of meeting eligibility criteria. The study will evaluate both effectiveness and implementation feasibility across tertiary paediatric and general hospitals in metropolitan and rural/regional settings. This paper presents the study protocol.

**Methods:**

Participating sites will select context‐appropriate interventions to support guideline‐concordant prescribing, informed by shared learning during the prospective phase. Effectiveness will be assessed using prospective audits, with key outcomes including timely guideline‐concordant IV‐to‐oral switch and hospital length of stay. Implementation feasibility will be evaluated using the Consolidated Framework for Implementation Research through surveys of local project committee members conducted before and after 12 months of audit and feedback.

**Conclusions:**

This study will assess the effectiveness and feasibility of translating and scaling best‐practice IV‐to‐oral antibiotic switch guidelines for children in real‐world hospital settings.

## 1 Introduction

Approximately half of hospitalised children in Australia receive systemic antibiotics [1]. Intravenous (IV) antibiotics are commonly used in paediatric inpatient care and, compared with oral therapy, may result in higher administration costs, pain, distress and discomfort, prolonged hospitalisation and risks associated with vascular access and line‐related complications [1, 2, 3, 4, 5, 6]. Many children receiving IV antibiotics could be treated effectively with oral therapy, enabling earlier hospital discharge and reducing treatment‐related burden for children and their families. This shift to oral treatment has the potential to improve health‐related quality of life, reduce healthcare costs and trauma, boost patient and family satisfaction and ease the strain on healthcare resources [7].

The Australasian Stewardship of Antimicrobials in Paediatrics (ANZPID‐ASAP) group developed guidelines for IV‐to‐oral antibiotic switch based on evidence generated through a systematic review, published in 2016 [8]. A subsequent pilot study at Sydney Children's Hospital, Randwick (SCH), successfully implemented these guidelines, significantly increasing the timely switch (defined as within 24 h of being eligible for the switch to oral antibiotics, according to endorsed guidelines [8]) from IV to oral antibiotics from 64% to 82% and reducing the median hospital length of stay by 18%, with no significant increase in adverse safety outcomes [3].

Whilst the pilot study demonstrated that implementation of evidence‐based IV‐to‐oral switch guidelines could improve prescribing practices within a specialist paediatric hospital, important questions remain regarding scalability, generalisability and implementation across diverse healthcare settings. Hospitals vary considerably in their patient populations, workforce composition, antimicrobial stewardship resources, access to paediatric infectious diseases expertise and local prescribing practices. Consequently, interventions shown to be effective in a single tertiary paediatric centre cannot be assumed to achieve similar outcomes elsewhere. HOMEFREE therefore extends previous work by evaluating implementation across multiple metropolitan, regional and rural hospitals, including both tertiary paediatric and non‐tertiary centres with varying levels of paediatric infectious diseases and antimicrobial stewardship support, whilst also examining the contextual factors that influence successful adoption and sustainability.

## 2 Aim

The current study, called the HOMEFREE study, aims to evaluate the effectiveness, implementation feasibility and scalability of promoting IV‐to‐oral antibiotic switch in hospitalised children using ANZPID‐ASAP guidelines [8] across selected hospitals in NSW, including tertiary paediatric, regional and non‐tertiary settings with varying levels of paediatric infectious diseases support. The primary outcome will be the proportion of eligible paediatric patients transitioned to oral therapy or antibiotic cessation within 24 h of meeting criteria, which will be assessed as primary outcome. Secondary objectives include assessing changes in switch timing, hospital length of stay, IV antibiotic duration, IV‐related complications and readmissions within 7 days. The study also examines feasibility and generalisability of implementation, considering interventions undertaken, barriers and enablers across sites.

## 3 Primary Hypothesis

The primary hypothesis for this study is that implementation of an IV‐to‐oral antibiotic switch programme, based on endorsed guidelines and using audit and feedback, will increase the proportion of hospitalised children transitioned to oral therapy or antibiotic cessation within 24 h of meeting eligibility criteria.

## 4 Methods and Analysis

### 4.1 Study Design

This study employs a hybrid type 2 effectiveness‐implementation design [9] to evaluate both the effectiveness and feasibility of evidence‐based IV‐to‐oral antibiotic switch guidelines in hospitalised children. Adapting methodology from a prior successful pilot study [3], this scaling study extends an educational audit and feedback approach to selected tertiary paediatric and non‐tertiary hospitals in New South Wales, Australia, encompassing metropolitan and rural/regional settings.

Implementation will be guided by the Model for Improvement framework, using Plan‐Do‐Study‐Act (PDSA) cycles to support iterative, site‐specific quality improvement. Effectiveness evaluation is detailed below. Implementation outcomes—acceptability, adoption, feasibility, fidelity and coverage—will be assessed in accordance with the Consolidated Framework for Implementation Research (CFIR) [10]. The study will be reported in accordance with the StaRI (Standards for Reporting Implementation Studies) statement [11] (Figure 1).

**FIGURE 1:**
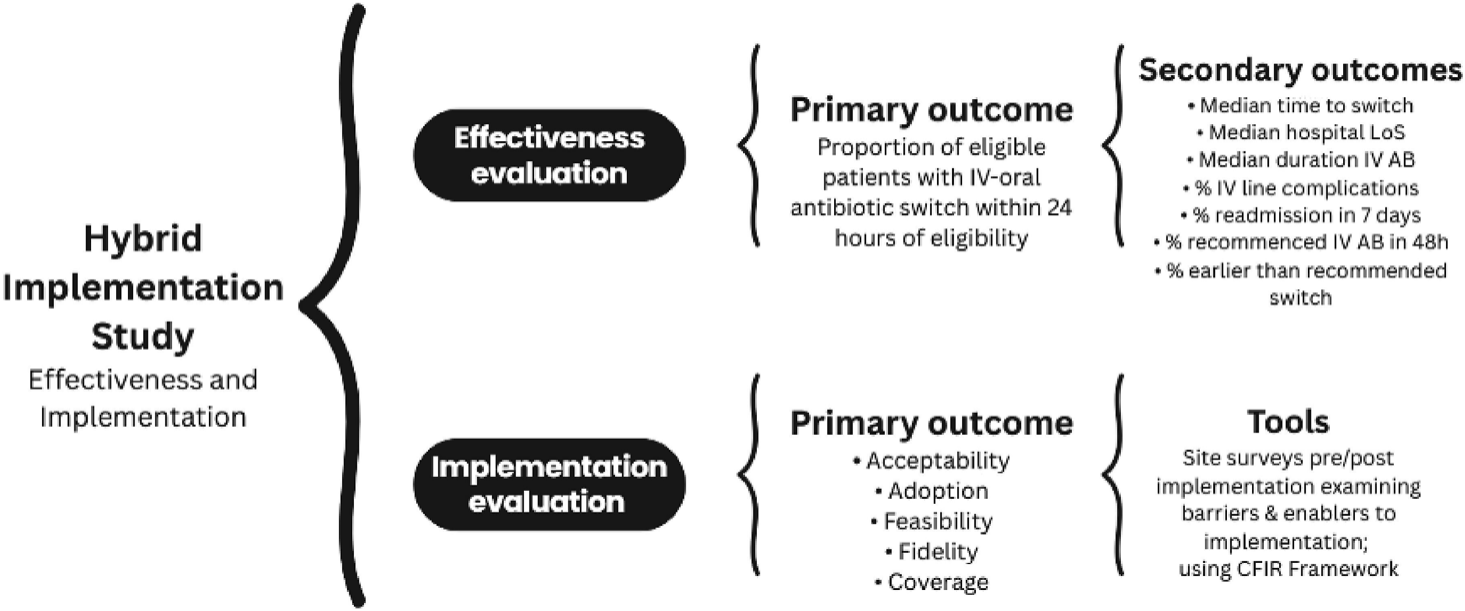
Study design and outcomes. AB, antibiotics, CFIR, Consolidated Framework for Implementation Research; IV, intravenous, LoS, length of hospital stay.

### 4.2 Study Procedures

#### 4.2.1 Site Recruitment and Selection

SCH, the site of the original pilot study, will serve as the lead site. Additional hospitals will be selected through an expression‐of‐interest (EOI). EOIs will include hospital location, capacity and anticipated recruitment volumes. The central Steering Committee will assess submissions to ensure diversity and adequate sample size across sites.

#### 4.2.2 Participants and Interventions

The primary clinical population is children under 18 years admitted to participating hospitals and treated with IV antibiotics for conditions specified in the ANZPID‐ASAP IV‐to‐Oral Switch Guidelines [8]. These patients form the basis for outcome measurement.

The core intervention is the dissemination and implementation of a programme promoting evidence‐based IV‐to‐oral antibiotic switch, using the ANZPID‐ASAP guidelines, supported by educational resources and a standardised implementation toolkit (shared files and tools are listed in the Supporting Information Appendix). Each participating hospital site will establish a multidisciplinary committee to lead local adaptation and delivery of the intervention. Sites are free to determine the composition of these from amongst paediatrics, infectious diseases, pharmacy, quality improvement and other site‐determined relevant stakeholders.

Effectiveness audit will be done via a standardised tool provided as an Excel spreadsheet (for local use) with deidentified audit results entered into a REDCap database (Supporting Information Appendix). Sites will be asked to collect retrospective audit data for 50 patients in the prior 12 months as baseline data and to familiarise auditors with the tool prior to beginning the programme and collecting data prospectively (up to five patients per week). This information will be reviewed by site committees and used, alongside local knowledge and observations, to deliver three to four PDSA cycles over 12 months, aiming to improve IV‐oral switch or other aspects of antimicrobial use identified via the audit progress. Sites may also incorporate additional measures to support early discharge or earlier transfer to care in home‐based settings, depending on local capacity and context. During the prospective PDSA cycles, site principal investigators and auditors will meet online at least quarterly to share experiences, barriers and enablers and allow ideas to be disseminated between sites.

#### 4.2.3 Sample Strategy

The sampling extent is pragmatic and informed by the prior pilot study [3]. In that study, 50 baseline patients and 249 prospective patients were reviewed. The percentage of children switched within 24 h of eligibility significantly increased from 32/50 (64%) at baseline to 203/249 (82%) post‐implementation (*p* = 0.006). For the current study, a minimum combined sample of 588 patients (294 baseline and 294 prospective) across all sites is estimated to provide 80% power to detect a 10% improvement in switch rates (*α* = 0.05). Given the number and diversity of participating hospitals, the total sample size is expected to exceed this target, with proportionately higher recruitment anticipated from sites with larger paediatric caseloads.

#### 4.2.4 Patient and Public Involvement

Patients and members of the public were not involved in the design or conduct of the pilot study that informed this protocol. However, patient and caregiver perspectives form an important component of the broader HOMEFREE programme. A concurrent consumer co‐design study is being undertaken with parents and caregivers of hospitalised children to better understand their experiences, information needs, preferences and perceived barriers and enablers related to IV‐to‐oral antibiotic switch and discharge home. This work includes interviews, journey mapping and co‐design activities and will inform the development of patient‐centred resources and implementation strategies to support safe and effective transition from hospital to home. Findings from this study will be reported separately and used to inform future implementation and scale‐up activities.

### 4.3 Implementation

Implementation of HOMEFREE interventions will be undertaken by local site teams as described above. The focus of the implementation evaluation will be to understand factors influencing adoption, acceptability, feasibility, fidelity and sustainability across participating sites. Sites will adopt the Model for Improvement framework, using PDSA cycles to guide iterative testing and refinement of locally adapted strategies [12]. Interventions may include targeted education for prescribers, audit and feedback and updates to local policies and procedures. Implementation will be supported by a standardised toolkit comprising evidence‐based guidelines, educational materials and evaluation protocols (Supporting Information Appendix).

Each site will be assigned a dedicated part‐time research assistant to coordinate implementation activities. Responsibilities include supporting the site principal investigator, facilitating staff and patient education, promoting programme awareness and compiling progress reports. This person will generally be responsible for conducting audits, with support provided by the site principal investigator and central project coordinator (D.T.) and project lead (B.M.). As noted above, local results will be reviewed in group online meetings, with successful strategies shared across participating sites to promote collective learning.

Pre‐implementation readiness assessment (Supporting Information Appendix) will be conducted to identify site‐specific barriers, enablers and resource requirements. Findings will inform adaptation of toolkit materials and implementation plans. Particular attention will be paid to implementation supports required to facilitate successful IV‐to‐oral antibiotic switch, including caregiver education processes, pharmacy involvement, discharge support activities and other locally relevant resources. Post‐implementation evaluation will be via reports from site investigators on self‐assessed acceptability, adoption, feasibility, fidelity and coverage within their institution. These will be reviewed by the Central Steering Committee and synthesised to report overall results.

### 4.4 Effectiveness Evaluation

#### 4.4.1 Clinical Outcomes

The primary clinical outcome measure will be:

A. The proportion of eligible patients on IV antibiotics that are switched to oral therapy or for whom antibiotics are ceased within 24 h of eligibility.

Secondary outcome measures will include:

B. Median time taken to switch eligible patients to oral antibiotics.

C. Median hospital length of stay.

D. Median duration of IV antibiotic therapy.

E. Proportion of patients with IV‐associated complications, including IV‐line infections and other recorded line‐related complications.

Balancing/additional measures:

F. Proportion of patients with readmissions due to infection within 7 days of discharge.

G. Proportion of patients recommenced on IV antibiotics within 48 h after oral switch.

H. Proportion of patients identified who switched > 24 h earlier than the minimum duration of IV therapy recommended by these guidelines. This balancing measure will be used to identify potential unintended consequences of the intervention and monitor for transitions occurring substantially earlier than recommended by evidence‐based guidance. These cases will not be included in the primary analysis but will be analysed and reported separately.

Pre‐planned economic and quality‐of‐life outcome evaluation is also planned as future sub‐studies. These will be reported separately. Data relating to the number of peripheral IV insertions or cannulation attempts is not routinely collected in a standardised manner across participating sites and is therefore not included in the current audit framework.

#### 4.4.2 Implementation Evaluation

Implementation outcomes will be assessed using the Consolidated Framework for Implementation Research (CFIR) [13], focusing on acceptability, adoption, feasibility, fidelity and coverage. Pre‐implementation site assessments will be conducted to identify barriers and enablers, informing site‐specific adaptations. Sites will self‐assess using a standardised tool (Supporting Information Appendix) and report back to a Central Steering Committee. Post‐implementation surveys and interviews with site leads and local committee members will evaluate actual implementation experiences. Reporting of outcomes will follow Standards for Reporting Implementation Studies (StaRI) Statement guidelines, as applicable [11].

## 5 Ethics and Dissemination

This study has been approved by The Sydney Children's Hospitals Network Human Research Ethics Committee (2022/ETH01407) with local site‐specific governance approval at each site. The authors declare that the research presented in this manuscript adheres to the ethical principles outlined in The Sydney Children's Hospitals Network Human Research Ethics Committee. All procedures involving human participants were conducted in accordance with the ethical standards of Australia and the Declaration of Helsinki (1964), as revised in 2013.

## 6 Discussion

This study will examine the effectiveness and feasibility of a programme to translate and scale best practice guidelines for hospital IV‐to‐oral antibiotic switch in children into real‐world practice. Although pilot implementation of an IV‐to‐oral antibiotic switch programme at a single dedicated paediatric hospital was successful, it cannot be assumed that this will automatically translate to successful implementation in other hospitals in different settings, including metropolitan non‐paediatric hospitals and rural settings.

Unlike the previously published single‐centre implementation study, HOMEFREE has been specifically designed to evaluate scalability, generalisability and implementation across heterogeneous healthcare environments. The study includes metropolitan, regional and rural hospitals, as well as both specialist paediatric and non‐tertiary centres, representing a broader range of clinical contexts and resource availability. A particular strength is the inclusion of hospitals with varying access to paediatric infectious diseases expertise and antimicrobial stewardship infrastructure, allowing assessment of whether guideline‐concordant IV‐to‐oral transition practices can be successfully implemented beyond specialist paediatric centres. The study also incorporates a hybrid effectiveness‐implementation design, formal implementation evaluation using the Consolidated Framework for Implementation Research, site‐specific adaptation through PDSA cycles and structured cross‐site learning activities. Collectively, these features will provide important evidence regarding factors associated with successful implementation and broader dissemination. The primary clinical outcome, the proportion of eligible patients switched to oral antibiotics within 24 h of eligibility according to pre‐specified guidelines, will assess whether a quality improvement programme of educational and other interventions, combined with audit and feedback, can be improved over the course of a year. It is also conceptually simple and can be assessed by auditors across various hospital types.

The quantitative secondary clinical outcomes aim to provide an understanding of the broader impacts of guideline implementation. These outcomes are relevant to hospital clinicians, administrators and patients/families and include duration of IV therapy, hospital length of stay, adverse events and readmissions. Hospital length of stay is recognised as a multifactorial outcome and should not be interpreted in isolation as a measure of intervention success. In paediatric settings, safe transition to oral therapy may require additional supports including caregiver education, medication administration training, pharmacy counselling, play therapy involvement, discharge planning and coordination of community‐based care. Accordingly, length of stay is included as one indicator of broader system impact rather than a standalone measure of effectiveness.

The implementation evaluation will complement these clinical outcomes by exploring contextual factors, barriers, enablers and resource requirements that may influence successful adoption of IV‐to‐oral transition practices. This includes understanding the education, support strategies and service‐level factors required to facilitate oral medication administration and transition from hospital to home.

Whilst IV‐related complications are included as a secondary outcome, the study does not capture the number of peripheral IV insertions or cannulation attempts, as these data are not always routinely available in a standardised format across all participating hospitals.

Balancing measures have also been included to monitor for potential unintended consequences of implementation efforts. In particular, the study will identify instances where transition to oral therapy occurs substantially earlier than recommended by guideline‐based minimum IV treatment durations, ensuring that improvements in transition rates are not achieved at the expense of appropriate antibiotic management.

Importantly, a concurrent consumer co‐design study will provide insights into parent and caregiver experiences, information needs and preferences related to IV‐to‐oral antibiotic transition. These findings will complement the implementation evaluation by identifying patient‐centred factors that influence successful transition from hospital to home and informing future implementation strategies and educational resources.

The study will offer insights into the efficacy and safety of the transition process and offer insights to inform future guidelines and policy. These measures are also designed to facilitate subsequent assessment of cost‐utility, examining potential financial benefits for the healthcare system and caregivers. Planned future quality‐of‐life analyses will examine patient and caregiver perspectives, an important component of evaluating a quality‐improvement programme's success and impacts.

A key strength of this protocol is the concurrent evaluation of implementation feasibility alongside clinical effectiveness. This is critical to understanding not only whether clinical guidelines are effective, but also how, why and under what conditions they succeed or fail in real‐world healthcare settings. Despite strong evidence supporting timely IV‐to‐oral antibiotic switch, variation in uptake across hospitals remains a persistent challenge, often due to contextual barriers, resource limitations and differences in prescribing culture [3]. By incorporating implementation outcomes—such as acceptability, appropriateness and fidelity—this study recognises that successful translation of evidence into practice requires more than clinical efficacy. It demands a nuanced understanding of the environment and processes that support sustainable adoption, adaptation and scale‐up of interventions. Evaluating implementation in parallel with clinical and economic outcomes strengthens the applicability of findings and ensures that recommendations for wider dissemination are grounded in practical realities, thereby bridging the evidence‐to‐practice gap in paediatric antimicrobial stewardship.

This study of IV‐to‐oral antibiotic switch guideline implementation incorporates assessment of effectiveness, implementation feasibility and scalability across diverse healthcare settings. By combining measurable clinical outcomes with implementation science methodologies, the study seeks to understand not only whether evidence‐based switch practices are effective, but also how and under what circumstances they can be successfully adopted and sustained. This holistic approach facilitates robust evaluation of guideline impact to inform future policy and practice in paediatric antibiotic management.

## Author Contributions

J.S. led the drafting of the initial manuscript, contributed to its review and revision and approved the final version for submission. B.M. contributed to the conception and design of the study, secured funding, supported manuscript drafting, critically reviewed and revised the content and approved the final version for submission. D.T., A.B., C.B., K.T., E.B., L.J., G.H., P.B. and L.H. contributed to the conceptualisation, study design, execution, synthesis and interpretation of findings. They provided critical review and input on the manuscript and approved the final version for submission. G.M. oversaw the design of the costing analysis, critically reviewed and revised the manuscript content and approved the final version for submission.

## Funding

This work was supported by the Hospitals United for Sick Kids and an NHMRC‐funded Investigator Grant led by Dr. McMullan (2021/GNT2008632).

## Conflicts of Interest

The authors declare no conflicts of interest.

## Supporting information


**Data S1:** Supporting Information.

## Data Availability

The data that supports the findings of this study are available in the supplementary material of this article.
