# Supplementary material for: HOMEFREE: Scaling‐Up Evidence‐Based Guidelines for IV‐to‐Oral Antibiotic Switch in Hospitalised Children, an Implementation Study Protocol
Source: J Paediatr Child Health. 2026 Aug 5;62(10):1901–6. doi: 10.1111/jpc.70528 (PMC13671250; doi:10.1111/jpc.70528)
Supplement: Supplementary file 1 [file JPC-62-1901-s001.docx]

HOMEFREE: Supplementary Appendix

Table of Contents

[1.	HOMEFREE Organisational Readiness Assessment Tool	2](#_Toc222911288)

[2.	HOMEFREE IV-Oral Switch Audit Tool Data Fields and Definitions	6](#_Toc222911289)

[3.	HOMEFREE Data Auditing PowerPoint Presentation for Sites	8](#_Toc222911290)

[4.	Practical Materials to Aid IV-oral Antibiotic Switch	10](#_Toc222911291)

[A: Dosing and Conversion Table	10](#_Toc222911292)

[B: General Principles IV-Oral Antibiotic Conversion	12](#_Toc222911293)

[5.	Educational Materials – Links	13](#_Toc222911294)

## 1. HOMEFREE Organisational Readiness Assessment Tool

| **ON TRACK** | **IMPROVEMENT REQUIRED** | **AT RISK** | **COMPLETE** | **NOT APPLICABLE** |
| --- | --- | --- | --- | --- |
| Organisation currently on track to achieving a state of readiness to support the project effectively. | Potential for full program benefits not to be achieved without intervention, however opportunities exist to strengthen this area. | Current organisational state is likely to impact the successful outcome of the project. Significant work required in this area prior to undertaking project activities. | Evidence can be provided to support the organisational readiness in this area and for the project to operate effectively. | Outlined requirement is not applicable for this quarter. |

| **Indicator** | **Criteria** | **2023** | | | |
| --- | --- | --- | --- | --- | --- |
|  |  | **Q1** | **Q2** | **Q3** | **Q4** |
| **Organisation** | | | | | |
| Organisation support | There is documented executive level support for the project. |  |  |  |  |
| Governance | Program activities and outcomes will be reported to all relevant stakeholders. |  |  |  |  |
| Department support | There is support from relevant departments, e.g., pharmacy, e.g. HITH, infectious diseases, and paediatrics. |  |  |  |  |
| Antimicrobial stewardship | There is evidence of AMS policy in place at the site. |  |  |  |  |
| **People** | | | | | |
| Leadership | A project team, including staff from relevant departments at various levels, has been formed and are committed to remaining engaged throughout the project. |  |  |  |  |
| Stakeholders | Stakeholders from relevant departments at various levels are aware of and support the program. |  |  |  |  |
|  | Consumer representatives have been identified, where appropriate, and are engaged with the program. |  |  |  |  |
| Project leaders and champions | A project research coordinator (e.g. Pharmacist, Nurse) and site principal investigatory have been identified to support implementation and data collection at the site |  |  |  |  |
|  | There is a plan for regular communication between the central project coordinator at UNSW/SCH and the site research co-ordinator and principal investigator. |  |  |  |  |
| **Policy** | | | | | |
| Policies and procedures | IV-to-Oral Antibiotic Switch and associated guidelines are available at the site and any areas requiring local adaptation have been identified. |  |  |  |  |
|  | Existing policies regarding treatment of infections have undergone review with any changes required identified. |  |  |  |  |
|  | Clear documentation exists to facilitate discharge to HITH, if available. |  |  |  |  |
|  | Processes for patient readmission/recommencement of IV therapy, if required, are documented and available to site investigators and clinical teams. |  |  |  |  |
|  | Changes in local policies required are made available to the project team. |  |  |  |  |
| Existing training infrastructure | Policies to support clinician education at the site are available and have been identified |  |  |  |  |
|  | Project champions have received education on HOMEFREE and IV-to-Oral switch. |  |  |  |  |
|  | Patients and their carers/guardians are provided with education regarding reasons to return to hospital and contact information for relevant staff. |  |  |  |  |
| **Infrastructure** | | | | | |
| Electronic medical records | Electronic medical records are available to relevant staff. |  |  |  |  |
|  | Hard copy policy and program details are readily available if electronic systems are not. |  |  |  |  |
| Patient review | Adequate infrastructure for data collection is in place (including medical record, computer and internet access). |  |  |  |  |
| Monitoring | Capacity for the collection of data relating to key performance indicators for program efficacy and safety. |  |  |  |  |
|  | Electronic medical records systems are sufficient for collection of project data and performance indicators. |  |  |  |  |
| **Evaluation** | | | | | |
| Safety | Critical performance indicators relating to patient safety will be captured (hospital representation, readmission, missed infections, adverse events). |  |  |  |  |
|  | Formal analysis will be completed for all patients affected by adverse events or requiring readmission or representation in accordance with local policies, and relevant information provided to the project team as permitted. |  |  |  |  |
| Efficiency | Other performance indicators will also be monitored (proportion of patients switched, timeliness of switch, quality of life, length of stay in hospital, bed days saved.) |  |  |  |  |
| Quality of life | Capacity exists to obtain consent from patients and their carers/guardians for quality of life and health economics in a timely manner. |  |  |  |  |
| Economic outcomes | Required data can be collected to enable local health information systems to provide cost data. |  |  |  |  |
| Feedback to consumers  and staff | Processes for regular staff and consumer feedback have been identified, including capacity for reporting of performance indicators. |  |  |  |  |
| Feedback from consumers and staff | Opportunities exist and are utilised to process feedback from stakeholders regarding performance indicators. |  |  |  |  |
|  | Policy and education materials can be updated in response to feedback. |  |  |  |  |
| Ethics | Ethics and governance applications submitted to facilitate data collection. |  |  |  |  |

## 2. HOMEFREE IV-Oral Switch Audit Tool Data Fields and Definitions

| **AUDIT TOOL HEADERS** | **DATA DEFINITION** |
| --- | --- |
| **Study identifier** | A number corresponding to a given patient. A separate document should provide a key to match this number with the patient medical record number (MRN). Do NOT enter the MRN directly into any document that will be shared as part of this project. |
| **Date & time of admission** | The date/time that the patient is recorded as being admitted to hospital from ED, ward booking, etc. in the electronic medical record (eMR). |
| **Admitting speciality** | The speciality of the medical officer under which the patient was admitted to hospital. This does NOT include consulting departments who do not have primary care of the patient. |
| **Relevant diagnosis** | For which diagnosis included in the ANZPID Guidelines has this patient been treated? *Note:* some patients will have concomitant issues and the eMR may list them as having a different diagnosis. Please list the relevant diagnosis from the ANZPID Guidelines. |
| **Date & time IV medication commenced** | The time the patient received IV medication for the treatment of the diseases referred to in the ANZPID Guidelines, as per the eMR and/or pharmacy records. |
| **Date & time eligible for switch** | The date and time in which you have determined the patient was FIRST eligible, by reference to the criteria and eMR notes. |
| **Date & time oral antibiotic commenced** | The date and time in which you have determined the patient was switched to oral antibiotics or ceased IV medication, by reference to eMR notes and/or pharmacy data. The key factor is the time the decision was documented in eMR to switch, or documented in medication records, whichever is first. |
| **Time to switch (hours)** | This is calculated automatically - the difference between the time to actually switch and the time in which switch was first possible. |
| **Date & time of discharge** | The date and time the patient was discharged from acute care, including to HITH. |
| **Length of inpatient stay** | This is calculated automatically - the time between admission and acute discharge. |
| **Notes** | Space from comments to provide context or summarise relevant factors. Please note associated diagnoses, social factors, etc. that were relevant to admission. |
| **Primary IV antibiotic received** | The name of the primary drug used (NOT brand name) meaning the IV medication used to treat the diagnosis that appears in the ANZPID Guidelines. If more than one medication is used, this should be the definitive drug that is prescribed rather than empiric medication. If there is still more than one drug, please use the one for which the most doses were given and list the other in 'Notes'. |
| **Primary oral antibiotic received** | The name of the primary drug used (NOT brand name) meaning the oral medication used as a step-down drug in the treatment of the diagnosis that appears in the ANZPID Guidelines. If more than one medication is used, please use the one for which the most doses were given and list the other in 'Notes'. |
| **Readmission** | Was the patient readmitted for treatment of the same infection for which they were treated during their initial admission. This may include fulminant infections and those that have seeded, e.g., new metastatic foci judged to have been seeded from the initial infection. |
| **Line infection** | Did the patient experience an IV-line infection while in hospital? |

## 3. HOMEFREE Data Auditing PowerPoint Presentation for Sites

## 4. Practical Materials to Aid IV-oral Antibiotic Switch

These may be adapted by sites into posters and guidelines.

### A: Dosing and Conversion Table

| **IV antibiotic and dose** | **Suggested oral conversion** |
| --- | --- |
| Ampicillin/Amoxicillin  25-50 mg/kg/dose 6-hourly | Amoxicillin  15-30 mg/kg/dose 8-hourly |
| Benzylpenicillin  30-60 mg/kg/dose 6-hourly | Amoxicillin  15-30 mg/kg/dose 8-hourly  OR  Phenoxymethylpenicillin  10-12.5 mg/kg/dose 6-hourly |
| Cefotaxime  25-50 mg/kg/dose 6-to-8-hourly | Amoxicillin-Clavulanic acid  22.5 mg/kg/dose (amoxicillin component)  12-hourly     - For patients aged ≥2 months. For younger patients, seek ID advice.      - If treating *Pseudomonas* species or resistant Gram-negative infection, seek ID advice. |
| Ceftriaxone  50-100 mg/kg/dose 24-hourly |  |
| Piperacillin-Tazobactam  100 mg/kg/dose (piperacillin component) 6-to-8-hourly |  |
| Amoxicillin- Clavulanic acid  25mg/kg (amoxicillin component) 8-hourly |  |
| Ampicillin  25-50 mg/kg/dose 6-hourly  PLUS  Gentamicin/tobramycin  7.5 mg/kg/dose 24-hourly  PLUS  Metronidazole  12.5 mg/kg/dose 8-12hrly |  |
| Metronidazole  12.5 mg/kg/dose 8-12hrly | Metronidazole  10mg/kg 8-12hourly |
| Flucloxacillin  25-50 mg/kg/dose 6-hourly | Cefalexin  12.5-25 mg/kg/dose 6-hourly |
| Ciprofloxacin  10mg/kg/dose 12-hourly (8-hourly if severe) | Ciprofloxacin  10-15mg/kg/dose 12-hourly |
|   Vancomycin or clindamycin (all doses) | Seek ID advice    *Clindamycin or trimethoprim-sulfamethoxazole may be suitable* |
| These drugs have high bioavailability therefore **IV and PO doses are similar:** | |
| - Azithromycin - Trimethoprim-sulfamethoxazole - Clindamycin | - Linezolid - Moxifloxacin |

### B: General Principles IV-Oral Antibiotic Conversion

Suitable for lanyard cards, all sites have access to NSW health intranet sites, where the policy is housed.

## 5. Educational Materials – Links

These are provided as online resources and can also be used in local posters and lanyard cards.

A. **Antibiotic duration and timing of the switch from intravenous to oral route for bacterial infections in children: systematic review and guidelines,** McMullan, Brendan J et al, The Lancet Infectious Diseases, Volume 16, Issue 8, e139 - e152: <https://doi.org/10.1016/S1473-3099(16)30024-X>
B. **ANZPID-ASAP Guidelines for Antibiotic Duration and IV-Oral Switch in Children**: accessed 25 Feb 2026, available at: <https://media.starship.org.nz/anzpid-asap-guidelines-for-antibiotic-duration-and-iv-oral-switch-in-children/IV_Oral_Table_Alone.pdf>
C. **Changing from Intravenous to Oral Antibiotics: Information for Parents and Carers**: accessed 25 Feb 2026, available at: <https://www.cec.health.nsw.gov.au/keep-patients-safe/medication-safety/antimicrobial-stewardship/iv-to-oral-antibiotic-switch/information-for-parents-and-carers>
